# Associations of dietary and sedentary behaviours of pregnant women with their children’s birth weight: findings from the CHAT trial in Australia

**DOI:** 10.1017/S1368980023002161

**Published:** 2023-12

**Authors:** Huilan Xu, Limin Buchanan, Yong Wang, Philayrath Phongsavan, Louise A Baur, Li Ming Wen

**Affiliations:** 1 Health Promotion, Population Health Research & Evaluation Hub, Sydney Local Health District, Forest Lodge, NSW, Australia; 2 Sydney Institute for Women, Children and Their Families, Sydney Local Health District, Camperdown, NSW, Australia; 3 Sydney School of Public Health, Faculty of Medicine and Health, The University of Sydney, Sydney, NSW, Australia; 4 NHMRC Centre of Research Excellence in the Early Prevention of Obesity in Childhood (EPOCH), Sydney, NSW, Australia; 5 Charles Perkins Centre, The University of Sydney, Sydney, NSW, Australia; 6 Specialty of Child and Adolescent Health, Sydney Medical School, The University of Sydney, Sydney, NSW, Australia

**Keywords:** Birth weight, Maternal, Dietary behaviours, Sedentary time, Pregnancy

## Abstract

**Objective:**

To examine the associations of pregnant women’s dietary and sedentary behaviours with their children’s birth weight.

**Design:**

Secondary data analysis was conducted using data from a randomised controlled trial, Communicating Healthy Beginnings Advice by Telephone, conducted in Australia. Information on mothers’ socio-demographics, dietary and sedentary behaviours during pregnancy was collected by telephone survey at the third trimester. Birth weight data were extracted from the child’s health record book. Multinomial logistic regression models were built to examine the associations of pregnant women’s dietary and sedentary behaviours with children’s birth weight.

**Setting:**

Participating families.

**Participants:**

Pregnant women and their children.

**Results:**

A total of 1132 mother–child dyads were included in the analysis. The majority of infants (87 %, *n* 989) were of normal birth weight (2500 g to <4000 g), 4 % (*n* 50) had low birth weight (<2500 g) and 8 % (*n* 93) had macrosomia (≥4000 g). Mothers who ate processed meat during pregnancy were more likely to have macrosomia (adjusted risk ratio (ARR) 1·80, 95 % CI (1·12, 2·89)). The risk of macrosomia decreased as the number of dietary recommendations met by mothers increased (ARR 0·84, 95 % CI (0·71, 0·99)). Children’s birth weight was not associated with mothers’ sedentary time. Children’s low birth weight was not associated with mothers’ dietary and sedentary behaviours during pregnancy.

**Conclusion:**

Maternal consumption of processed meat during pregnancy was associated with an increased risk of macrosomia. Increasing number of dietary recommendations met by mothers was associated with a lower risk of macrosomia. The findings suggested encouraging pregnancy women to meet dietary recommendation will benefit children’s birth weight.

Unfavourable birth weight, as characterised by either low birth weight (<2500 g) or macrosomia (>4000 g), accounts for approximately 20 % of births worldwide^(1)^. Low birth weight is more prevalent in low- and middle-income countries and especially prevalent among vulnerable populations (lower socio-economic status)^(2)^. The prevalence of macrosomia has increased markedly in the past few decades especially among high-income countries and women with increased maternal weight and gestational weight gain^(3)^. Both low birth weight and macrosomia are associated with infant morbidity and mortality, as well as increased risk of poor health status later in life^(4)^. For example, low birth weight is linked to increased risks of non-communicable diseases such as cardiovascular disease and diabetes, while macrosomia is associated with increased risks of obesity and cancers in adulthood^(2,4)^. Mothers’ health behaviours during pregnancy, such as their dietary intake and activity levels, may influence fetal development and be an important modifiable determinant of healthy birth weight^(5,6)^.

Pregnancy is a critical time for caution about diets. Maternal dietary habits can have a direct effect on the growing fetus^(7)^ and subsequently the outcomes of pregnancy^(8)^. However, findings from previous research on the relationship between maternal dietary intake and birth weight remain inconclusive. A Norwegian study of 65 904 mother–child dyads linked poor diet to increased risks of both low birth weight and macrosomia^(1)^. Two studies, one from Japan (803 infant–mother dyads) and the other from Denmark (44 612 infant–mother dyads), found that a low-quality diet was associated with an increased risk of low birth weight^(9,10)^. These findings contrast with a US study of forty-one infant–mother dyads, which suggested that poorer diet quality may be related to higher birth weight^(11)^. Another study, from the USA, found no association between maternal dietary intake and birth weight^(12)^.

Another health behaviour during pregnancy that may influence birth weight is sedentary behaviour, which is mainly attributed to prolonged screen time. With an escalation in the use of digital technologies over the last two decades, prolonged and excessive screen time has become a public health concern. The increased sedentary time during pregnancy is associated with higher levels of LDL-cholesterol in mothers and a larger newborn abdominal circumference. However, its association with child birth weight remains inconclusive^(6)^. A Japanese study showed that the prevalence of excessive mobile phone use was significantly higher among pregnant women compared with non-pregnant women^(13)^. A study conducted in Australia found over one-third of study participants (408 first-time mothers) spent more than 3 h/d on screens^(14)^. Similarly, a Chinese study, which included 2345 pregnant women, found more than a quarter of participants had prolonged television and computer viewing time (2 h or more/d), and more than three-fifths of participants reported prolonged mobile phone viewing time (1 h or more/d)^(15)^. The association between screen time during pregnancy and birth weight has, to our knowledge, only been addressed in a Japanese study, which suggested that excessive mobile phone use may be linked to an increased risk for low birth weight^(13)^.

A better understanding of the association between maternal dietary and sedentary behaviours during pregnancy and birth weight will inform future health promotion interventions. Research on the association between mothers’ dietary and sedentary behaviours and birth weight is scarce and results are inconclusive. To address this knowledge deficit, this study investigated whether mothers’ dietary and sedentary behaviours during pregnancy are associated with their children’s birth weight outcomes.

## Methods

### Study design

A secondary data analysis was conducted using the baseline data from a three-arm randomised controlled trial, Communicating Healthy Beginnings Advice by Telephone (CHAT)^(16)^, a health promotion intervention aimed at reducing early risk factors for childhood obesity. The CHAT study delivered an intervention targeting body mass index (BMI), eating and screen time behaviours of children in the first years of life. Participants were assigned into telephone, short message service or control arms. Staged interventions were delivered for mothers from the antenatal stage (third trimester of pregnancy) until children were aged 2 years. The CHAT randomised controlled trial was conducted across four local health districts in New South Wales (NSW), Australia: Sydney, South Eastern Sydney, South Western Sydney and Southern NSW. The trial was approved by the Sydney Local Health District Ethics Review Committee (Protocol No. X16–0360 and LNR/16/RPAH/495). Further details of the CHAT randomised controlled trial’s protocol have been published elsewhere^(16)^.

### Participants

**Fig. 1:**
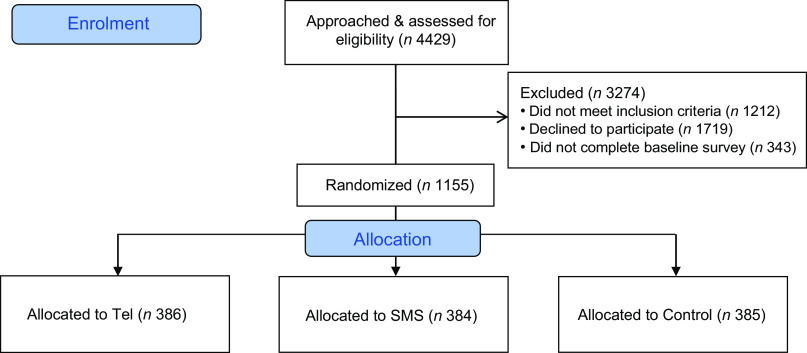
Recruitment process

Pregnant women in their third trimester (28–34 weeks) were recruited from eight hospital sites within the above four districts between February and July 2017. Potential participants were approached by research assistants with participant information sheet at the antenatal clinics and invited to participate in the study. Women were eligible to participate in the study if they were 16 years or above, between 28 and 34 weeks pregnant, capable to communicate in English, owned a mobile phone device and resided in the recruitment areas. Women were excluded if they had a severe medical condition, were expecting multiple births or with babies with known major fetal anomalies. Written consent was obtained from each participant. Participants were then required to fill in a registration form to provide their contact details for baseline data collection. The recruitment process was shown in Fig. 1. Details of the CHAT study recruitment and participants have been published elsewhere^(17)^.

### Measures and data collection

Data collection was conducted by a market survey company using the computer-assisted telephone interview. All data were collected at baseline when mothers were 28–34 weeks pregnant except the birth weight and child sex data. Data on child birth weight and sex were collected when mothers completed the telephone survey at 6 months post-partum.

#### Child birth weight

Mothers were asked to provide the birth weight and child sex noted in the My Personal Health Record (the ‘Blue Book’), which was issued to all parents in NSW when their child was born. The newborn birth weight was measured and recorded together with other birth data in the Blue Book^(18)^. The birth weight was grouped into three categories based on the common birth weight cut-off points: low birth weight (<2500 g), normal birth weight (2500 to <4000 g) and macrosomia (≥4000 g)^(2,19)^.

#### Mothers’ dietary and sedentary behaviours

Dietary behaviour was assessed using questions derived from the NSW Adult Population Health Survey, which was an annual cross-sectional telephone-based survey of residents aged 16 years and older living in NSW^(20,21)^. The validity and reliability of the survey questions have been demonstrated in adult population in NSW^(22)^ and have previously been used to examine dietary behaviours among pregnant women^(14,23)^. Mothers were asked about their frequency of processed meat, fast food and potato crisps intake (times per day or week or month). Responses were recorded as times per day/per week/per month, rarely/never and don’t know/refused. Responses were further dichotomised into ‘Yes’ and ‘No’, where ‘No’ referred to rarely/never based on the diet recommendation for pregnant women (avoid proceed meat, fast food and potato crisps)^(24)^. The amounts of vegetables and fruit (serves per day or week) and soft drinks intake (cups per day or week or month) were also measured. Vegetable, fruit and soft drink consumptions were further categorised into two groups (Yes/No) based on whether the mother met the recommended daily consumption of vegetables (≥5 serves/d), fruit (≥2 serves/d) and soft drink (no) during pregnancy^(24)^. A continuous variable (ranging from 0 to 6) called ‘Dietary behaviour’ was generated based on the number of dietary recommendations the mother met. The higher the score, the better the dietary behaviour. Another continuous variable (ranging from 0 to 4) called ‘Junk Food’ was generated based on the number of ‘Junk Food’ items (processed meat, fast food, chips and soft drink) consumed by the mother. The higher the score, the worse the dietary behaviours (online Supplemental File).

The sedentary time question was derived from the NSW Adult Population Health Survey in 2015^(25)^. Participants were asked about their daily time of sitting. The mean and median screen time were both 5 h/d. Therefore, sedentary time was dichotomised into ‘≤5 h/d’ and ‘>5 h/d’. Dietary and sedentary time questions used in this survey are listed in the online Supplemental File.

#### Mothers’ demographics

Questions from the NSW Adult Population Health Survey^(20)^ were used to collect information on mothers’ age, country of birth, language spoken at home, education level, household income, marital and employment status, whether they were first-time mothers, pre-pregnancy weight and height, smoking status and gestational diabetes status (including pre-pregnancy diabetes). Mothers’ pre-pregnancy weight and height data were converted into pre-pregnancy BMI (kg/m^2^). Mothers were grouped into four categories based on their pre-pregnancy BMI: <18·5 kg/m^2^ (underweight), healthy weight (18·5 to <25 kg/m^2^), overweight (25·0 to <30 kg/m^2^) or obesity (≥30 kg/m^2^)^(26)^. Gestational age was estimated based on mothers’ self-reported weeks pregnant on the day they were recruited, and the child’s date of birth collected at the 6 months telephone survey.

### Statistical analysis

Statistical analysis was performed using Stata software, version 16 (StataCorp LP 2016). Statistical significance was set at a *P* value <0·05 for all statistical tests, and testing was two-sided. Mothers’ demographics and their dietary and sedentary behaviours were presented in numbers and percentages by three birth weight groups (low, normal and macrosomia). They were compared across the birth weight status by using Chi-squared tests. Multinomial logistic regression models were built to investigate whether children’s birth weight status was associated with mothers’ dietary and sedentary behaviours during pregnancy. Chi-squared and Pearson’s correlation tests were conducted to assess the multicollinearity between the variables before adding them into the regression model. Since the CHAT study was a randomised controlled trial and the intervention started at late pregnancy, in order to take intervention effect into account, all multinomial logistic regression models were adjusted for intervention allocation. Based on previous literature^(23,27)^, maternal age, country of birth, language spoken at home, pre-pregnancy BMI, first-time mother, gestational diabetes during pregnancy, smoking status during pregnancy, infant sex and gestational age were considered as potential confounding factors. Backward elimination approach was used to identify confounding factors. All potential confounding factors were included in a multinominal logistic regression model. The least significant variables were progressively dropped until only those with *P* < 0·05 remained. Adjusted risk ratios and the 95 % CI were reported. To test the robustness of the association between maternal dietary and sedentary behaviour during pregnancy and children’s birth weight status, sensitive analysis were conducted by comparing the relative risk ratios for low birth weight or macrosomia between bivariate and multinomial logistic regression models. Chi-squared test for trend was used to examine whether the risk of low birth weight or macrosomia decreased or increased with increasing the number of dietary recommendations met by mothers and ‘Junk Food’ items consumed by mothers.

## Results

Baseline data were available for 1155 women. A total of 1132 birth weight data were obtained and included in this current study. Maternal characteristics are shown in Table 1. Sixty-three percentage of mothers were born overseas, predominantly in Asia, approximately 54 % of mothers spoke English at home, 61 % had normal pre-pregnancy BMI, 97 % were non-smokers and 68 % did not have diabetes (including gestational diabetes). The mean birth weight was 3·32 kg (sd 0·51, range: 1·15–4·90 kg). The majority of infants were of normal birth weight (87 %), with only 5 % of low birth weight and 8 % having macrosomia. There were significant differences in mothers’ country of birth, language spoken at home, pre-pregnancy BMI, diabetes and being a first-time mother among children’s birth weight status. Mean and median gestational age were both 39 weeks.

**Table 1:** Mothers’ baseline characteristics by infant birth weight status

Variables	Total		Low birth weight		Normal		Macrosomia		*P*
	*n*	%	*n*	%	*n*	%	*n*	%	
Age (years)									0·713
<30	358	32	14	28	317	32	27	29	
≥30	774	68	36	72	672	68	66	71	
Country of birth									0·043
Overseas	711	63	35	70	628	63	48	52	
Australia	421	37	15	30	361	37	45	48	
Language spoken at home									<0·0001
Other	519	46	34	68	462	67	23	25	
English	613	54	16	32	527	53	70	75	
Household income									0·655
<$80 000	379	38	20	43	327	37	32	36	
≥ $80 000	630	62	26	57	546	63	58	64	
Marital status									0·121
Other	78	7	0	0	70	7	8	9	
Married/de-facto partner	1053	93	50	100	919	93	84	91	
Employment status									0·794
Others	434	38	17	34	382	39	35	38	
Employed	697	62	33	66	606	61	58	62	
Education									0·332
Up to HSC* to TAFE†/Diploma	383	34	17	34	328	33	38	41	
University/tertiary	747	66	33	66	659	67	55	59	
First-time mother									0·036
No	521	46	18	36	450	46	53	57	
Yes	611	54	32	64	539	54	40	43	
Pre-pregnancy BMI									0·009
Normal	676	61	26	52	603	62	47	52	
Underweight	44	4	3	6	41	4	0	0	
Overweight	232	21	13	26	198	21	21	23	
Obesity	154	14	8	16	123	13	23	25	
Smoking status									0·778
Yes	38	3	2	4	34	3	2	2	
No	1094	97	48	96	955	97	91	98	
Diabetes (including gestational)									0·018
Yes	367	32	25	50	316	32	23	28	
No	765	68	25	50	673	68	67	72	
Intervention allocation									0·231
Telephone support	370	33	17	34	327	33	26	28	
SMS support	382	34	19	38	337	34	26	28	
Control	380	33	14	28	325	33	41	44	

	Mean	sd	Mean	sd	Mean	sd	Mean	sd	
Gestational age (weeks)	39	1·72	35·3	2·55	39·1	1·24	39·8	1·34	<0·0001

SMS, short message service.

* HSC, higher school certificate (Year 12).

† TAFE, technical and further education.

*P, P*-value of Chi-squared test.

Maternal dietary and sedentary behaviours by child’s birth weight status are presented in Table 2. Most mothers met dietary recommendations for fruit (≥2 serves/d, 71 %) and soft drink (no soft drink, 63 %). More than half of mothers (60 %) had sedentary time less than the average (5 h/d). Less than half of mothers met dietary recommendations for fast food (38 %), processed meat (48 %) and chips (25 %). Only 9 % of mothers met recommendation for vegetable (5 serves per day). While there were no significant differences in mothers’ fruit, vegetable, soft drink, fast food and sedentary time among children’s birth weight status, there were significant differences in mothers’ consumption of processed meat and chips among children’s birth weight status. Higher proportion of mothers who ate processed meat and chips were observed in the macrosomia group. Although, overall, the differences in mothers’ fast food consumption among children’s birth weight status were not significant, higher proportion of mothers who ate fast food was observed in the macrosomia group.

**Table 2:** Mothers’ dietary and sedentary behaviours during third trimester by infant birth weight status

Variables	Total		Low birth weight		Normal		Macrosomia		*P*
	*n*	%	*n*	%	*n*	%	*n*	%	
Fruit									0·874
<2 serves/day	326	29	16	32	283	29	27	29	
≥2 serves/day	806	71	34	68	706	71	66	71	
Vegetable									0·259
<5 serves/day	1028	91	44	88	903	92	81	87	
≥5 serves/day	101	9	6	12	83	8	12	13	
Soft drink									0·201
No	711	63	34	68	626	63	51	55	
Yes	421	37	16	32	363	37	42	45	
Fast food									0·110
No	428	38	18	36	384	39	26	28	
Yes	702	62	32	64	603	61	67	72	
Processed meat									0·014
No	540	48	26	52	483	49	31	33	
Yes	592	52	24	48	506	51	62	67	
Chips									0·009
No	288	25	18	36	257	26	13	14	
Yes	844	75	32	64	732	74	80	86	
Sedentary time									0·874
≤5 h/d	677	60	29	58	590	60	58	62	
>5 h/d	443	40	21	42	387	40	35	38	

*P*, *P*-value of Chi-squared test.

Results from multinomial logistic regression analyses are presented in Table 3. Mothers who ate fast food (risk ratio (RR) 1·64, 95 % CI (1·03, 2·63)), processed meat (RR 1·91, 95 % CI (1·22, 2·99)) and chips (RR 2·16, 95 % CI (1·18, 3·95)) were more likely to have macrosomia than children with normal birth weight. However, after adjusting for confounders, macrosomia was only associated with mothers’ processed meat consumption. The macrosomia RR was attenuated by 6 %. Compared with mothers who did not eat processed meat, mothers who ate processed meat were more likely to have infants with macrosomia than normal birth weight with adjusted risk ratio 1·80 (95 % CI (1·12, 2·89)). Nevertheless, macrosomia was associated with mothers’ ‘Junk Food’ consumption (expressed as the number of four ‘Junk Food’ items – soft drink, fast food, processed meat, and chips the mother consumed) after adjusting for confounders. For every increase in the items of ‘Junk Food’ consumed by a mother, a 31 % increase in the relative risk of macrosomia would occur (adjusted risk ratio 1·31, 95 % CI (1·07, 1·60)). The Chi-squared test for trend also showed a strong evidence that the risk of macrosomia increased with increasing the items of ‘Junk Food’ consumed by a mother (*P* < 0·0001). When examining mothers’ whole diet, macrosomia was associated with mothers’ dietary behaviour (expressed as the number of dietary recommendations met by the mother) after adjusting for confounders. For every increase in the number of dietary recommendations met by a mother, a 16 % reduction in the relative risk of macrosomia would occur (adjusted risk ratio 0·84, 95 % CI (0·71, 0·99)). The Chi-squared test for trend also showed that the risk of macrosomia decreased with an increasing number of dietary recommendations met by a mother (*P* = 0·002). For mothers’ ‘Junk Food’ consumption and dietary behaviour, the macrosomia RR was attenuated by 7 % and 5 % after controlling for confounders. There were no significant associations between children’s birth weight status and mothers’ sedentary time. And children’s low birth weight was not associated with mothers’ dietary and sedentary behaviours during pregnancy.

**Table 3:** Risk of low birth weight and macrosomia by mothers’ dietary and sedentary behaviours during third trimester

Variables	Low birth weight *v*. normal birth weight						Macrosomia *v*. normal birth weight					
	RR	95 % CI	*P*	ARR	95 % CI	*P*	RR	95 % CI	*P*	ARR	95 % CI	*P*
Fruit			0·606			0·868			0·932			0·936
<2 serves/day	Ref			Ref			Ref			Ref		
≥2 serves/day	0·85	0·46, 1·57		1·07	0·47, 2·42		0·98	0·61, 1·57		1·02	0·62, 1·68	
Vegetable			0·381			0·692			0·148			0·339
<5 serves/day	Ref			Ref			Ref			Ref		
≥5 serves/day	1·48	0·61, 3·58		1·30	0·35, 4·85		1·61	0·84, 3·08		1·39	0·71, 2·75	
Soft drink			0·501			0·457			0·108			0·387
No	Ref			Ref			Ref			Ref		
Yes	0·81	0·44, 1·49		0·74	0·33, 1·64		1·42	0·93, 2·18		1·23	0·77, 1·95	
Fast food			0·681			0·199			0·039			0·136
No	Ref			Ref			Ref			Ref		
Yes	1·13	0·62, 2·05		1·66	0·76, 3·62		1·64	1·03, 2·63		1·45	0·89, 2·38	
Processed meat			0·663			0·654			0·005			0·016
No	Ref			Ref			Ref			Ref		
Yes	0·88	0·50, 1·56		1·18	0·57, 2·44		1·91	1·22, 2·99		1·80	1·12, 2·89	
Chips			0·120			0·285			0·012			0·054
No	Ref			Ref			Ref			Ref		
Yes	0·62	0·34, 1·13		0·66	0·31, 1·41		2·16	1·18, 3·95		1·84	0·99, 3·44	
Junk food	0·91	0·72, 1·14	0·400	1·00	0·75, 1·34	0·994	1·41	1·16, 1·70	<0·0001	1·31	1·07, 1·60	0·009
Dietary behaviour	1·08	0·88, 1·32	0·451	1·02	0·78, 1·33	0·895	0·80	0·68, 0·93	0·005	0·84	0·71, 0·99	0·047
Sedentary time			0·736			0·900			0·709			0·815
≤5 h/d	Ref			Ref			Ref			Ref		
>5 h/d	1·10	0·62, 1·96		1·05	0·50, 2·21		0·92	0·59, 1·43		0·95	0·60, 1·50	

RR, risk ratio; ARR, adjusted risk ratio. Adjusted for mothers’ language spoken at home, pre-pregnancy BMI, diabetes (including gestational diabetes), gestational age, infant sex and intervention allocations.

Junk food, processed meat, fast food, chips and soft drink, ranging from 0 to 4.

Dietary behaviour, number of dietary recommendations met by mothers, ranging from 0 to 6.

## Discussion

In this study, we found that mothers’ consumption of processed meat during late pregnancy was associated with macrosomia. Although no statistically significant associations were found between children’s birth weight status and mothers’ fruit, vegetable, soft drink, fast food and chips consumptions, the risk of macrosomia decreased with increasing the number of dietary recommendations met by a mother. These findings suggest that encouraging mothers to meet more dietary recommendations, especially avoiding processed meat during pregnancy, may reduce the chance of having high birth weight.

The study also showed the risk of having macrosomia increased with an increase in the items of ‘Junk Food’ consumed by the mother. This finding is aligned with another Australian study on 368 first-time mothers showing maternal ‘junk food’ diet increased the risk of having a macrosomic infant^(23)^. Similarly, a Brazilian longitudinal study analysing 1298 pregnant women also found a positive association between salty snacks (i.e. food with high concentration of simple carbohydrates, fats and low amounts of protein and micronutrients) and high birth weight^(27)^.

The current study did not find associations of maternal fruit and vegetable intakes with their child’s birth weight. These findings are aligned with a systematic review, which found inconclusive evidence of a specific protective effect of fruit and vegetable consumption on infant birth weight^(5)^. In contrast, a large-scale prospective cohort study among pregnant Norwegian women found protective effects of diets comprising vegetables, fruit and water on birth outcomes^(28)^. Similarly, a study among Danish pregnant women found a small but statistically significant positive association between fruit and vegetable consumption and birth weight^(29)^. It should be noted that only 9 % of our study participants met the recommended vegetable consumption, and hence the association between vegetable consumption and child birth weight could be attenuated since the majority of the participants did not meet the dietary recommendations.

The current study found that more than half of the mothers did not meet the dietary recommendations for vegetable (≥5 serves/day), fast food (no), processed meat (no) and chips (no) during pregnancy. Although the majority (91 %) did not meet the recommendation for vegetables, more than half reported having 2–4 serves of vegetables per day. The vegetable consumption of our study participants was comparable with another study in Australia that found only 10 % of pregnant women met the recommendation, with a median of 2 serves per day^(30)^. The proportion of mothers who ate processed meat was also much less in our study (52 %) than the previous study (89 %)^(30)^. In the current study, 32 % of women had diabetes (including gestational diabetes). This was higher than the Australia wide data (16·1 % in 2017–2018)^(31)^, which may be attributed to the high percentage of women from Asian backgrounds^(32)^. Regarding the birth weight status, the rate of macrosomia (8 %) and low birth weight (5 %) in the current study was comparable to the population data in NSW in 2017 at 9·5 % and 6·7 %, respectively^(33)^.

In this study, we did not find associations between mothers’ sedentary time and children’s birth weight status. In contrast to the data on maternal physical activity and pregnancy outcomes, research on association between sedentary behaviours and birth weight is scarce and with conflicting findings. Three studies found no association between sedentary behaviour during pregnancy and birth weight^(34–36)^. One UK study found that sedentary behaviours (assessed by asking if participants mostly sitting during pregnancy) were inversely associated with infant size at birth^(37)^. A Japanese study also found that excessive mobile phone use during pregnancy was a significant predictor of lower birth weight^(13)^. In contrast, a cross-sectional study on 112 pregnant women in UK found increased sedentary time in third trimester was linked to macrosomia^(38)^. To our knowledge, there are no specific recommendations developed for sedentary time during pregnancy. Recommendations for pregnant women emphasise reducing sitting time and breaking up long periods of sitting or standing still^(39)^. More research on the association between sedentary time during pregnancy and birth outcomes is needed to inform guidelines for pregnant women.

### Strengths and limitations

The strengths of the current study are the large sample size and prospective design of the study. The prospective design, whereby dietary and sedentary behaviours were assessed before the outcome (birth weight), has minimised recall bias. To our knowledge, this is the first study that has examined the associations of maternal dietary and sedentary behaviours with birth weight in Australia. This current study contributes to an under-researched area.

However, this investigation has several limitations. First, gestational age data were estimated based on mothers’ self-reported weeks pregnant, which is subjected to estimation error. Birth weight is determined by both fetal growth and the duration of gestation^(40)^. And rapid gestational weight gain was also associated with macrosomia^(41)^. However, our study did not have access to the participants’ medical records to collect those data. Second, both the maternal dietary and sedentary behaviours were based on questionnaires reported by the pregnant women. Self-reported data, especially dietary habits, have often been criticised for their subjective nature and estimation error. Previous studies that investigated dietary habits have also showed that female participants tend to under-report their unhealthy food consumption in a way to portray themselves more favourably^(42)^. Although the questions used to measure dietary and sedentary behaviours were both derived from questionnaires that have previously been validated and widely used in adult populations^(22,25)^, and been used to assess health behaviours during pregnancy^(14,23)^, the questionnaire has not been specifically validated for use during pregnancy. Third, human diet is complex and the dietary questions may not capture the whole picture of dietary intake. For instance, alcohol intake during pregnancy that may influence birth weight^(45)^ was not assessed in the survey; the whole energy intake (the amount of food) was not assessed in the survey either. Additionally, the dietary questions predominantly assessed the Anglo-Celtic type of food and beverages. Cultural food preferences were not taken into consideration. Around 63 % of our study population were born outside of Australia. The survey may not have captured the overall dietary intake of some participants. Fourth, the survey only asked about time spent sitting on a weekday but not on a weekend day. Sedentary time may vary between weekend and weekdays. Therefore, mothers’ sedentary time we assessed may not reflect their sedentary time correctly. In our study, 40 % (*n* 443) mothers spent more than 5 h/d on sitting. Fazzi *et al.* assessed twenty-six studies on sedentary behaviours during pregnancy, with time spent in sedentary behaviours ranging from 7 to 18 h/d^(6)^. Lastly, both dietary and sedentary behaviours measures in the current study only captured a snapshot of the third trimester pregnancy; they may not reflect the dietary and sedentary behaviours during the entire pregnancy.

## Conclusion

Maternal dietary behaviours are independently associated with macrosomia. Our findings provide valuable insights for future health promotion interventions aiming to improve pregnancy outcomes by reducing mothers’ unhealthy dietary behaviours.

## Supplementary material

For supplementary material accompanying this paper visit https://doi.org/10.1017/S1368980023002161

## References

[1] Englund-Ögge L , Brantsæter AL , Juodakis J et al. (2019) Associations between maternal dietary patterns and infant birth weight, small and large for gestational age in the Norwegian mother and child cohort study. Eur J Clin Nutr 73, 1270–1282. 30459338 10.1038/s41430-018-0356-y PMC6760641

[2] WHO (2014) WHO Global Nutrition Targets 2025: Low Birth Weight Policy Brief. https://apps.who.int/iris/bitstream/handle/10665/149020/WHO_NMH_NHD_14.5_eng.pdf?sequence=2&isAllowed=y (accessed August 2022).

[3] Surkan PJ , Hsieh CC , Johansson ALV et al. (2004) Reasons for increasing trends in large for gestational age births. Obstet Gynecol 104, 720–726. 15458892 10.1097/01.AOG.0000141442.59573.cd

[4] Risnes KR , Vatten LJ , Baker JL et al. (2011) Birthweight and mortality in adulthood: a systematic review and meta-analysis. Int J Epidemiol 40, 647–661. 21324938 10.1093/ije/dyq267

[5] Murphy MM , Stettler N , Smith KM et al. (2014) Associations of consumption of fruits and vegetables during pregnancy with infant birth weight or small for gestational age births: a systematic review of the literature. Int J Women’s Health 6, 899–912. 25349482 10.2147/IJWH.S67130 PMC4208630

[6] Fazzi C , Saunders DH , Linton K et al. (2017) Sedentary behaviours during pregnancy: a systematic review. Int J Behav Nutr Phys Act 14, 32. 28298219 10.1186/s12966-017-0485-z PMC5353895

[7] Blumfield ML , Hure AJ , MacDonald-Wicks LK et al. (2012) Dietary balance during pregnancy is associated with fetal adiposity and fat distribution. Am J Clin Nutr 96, 1032–1041. 23034964 10.3945/ajcn.111.033241

[8] Kind KL , Moore VM & Davies MJ (2006) Diet around conception and during pregnancy – effects on fetal and neonatal outcomes. Reprod BioMed Online 12, 532–541. 16790095 10.1016/s1472-6483(10)61178-9

[9] Okubo H , Miyake Y , Sasaki S et al. (2012) Maternal dietary patterns in pregnancy and fetal growth in Japan: the Osaka maternal and child health study. Br J Nutr 107, 1526–1533. 21929833 10.1017/S0007114511004636

[10] Knudsen VK , Orozova-Bekkevold IM , Mikkelsen TB et al. (2008) Major dietary patterns in pregnancy and fetal growth. Eur J Clin Nutr 62, 463–470. 17392696 10.1038/sj.ejcn.1602745

[11] Grandy M , Snowden JM , Boone-Heinonen J et al. (2018) Poorer maternal diet quality and increased birth weight. J Matern-Fetal Neonatal Med 31, 1613–1619. 28514885 10.1080/14767058.2017.1322949 PMC5694379

[12] Poon A , Yeung E , Boghossian N et al. (2013) Maternal dietary patterns during third trimester in association with birthweight characteristics and early infant growth. Scientifica 2013, 786409. 24490111 10.1155/2013/786409 PMC3893866

[13] Lu X , Oda M , Ohba T et al. (2017) Association of excessive mobile phone use during pregnancy with birth weight: an adjunct study in Kumamoto of Japan environment and children’s study. Environ Health Prev Med 22, 52. 29165149 10.1186/s12199-017-0656-1 PMC5664573

[14] Wen LM , Simpson JM , Baur LA et al. (2011) Family functioning and obesity risk behaviors: implications for early obesity intervention. Obesity 19, 1252–1258. 21127478 10.1038/oby.2010.285

[15] Xu X , Liu D , Rao Y et al. (2018) Prolonged screen viewing times and sociodemographic factors among pregnant women: a cross-sectional survey in China. Int J Environ Res Public Health 15, 403. 29495439 10.3390/ijerph15030403 PMC5876948

[16] Wen LM , Rissel C , Baur LA et al. (2017) A 3-arm randomised controlled trial of communicating Healthy Beginnings advice by telephone (CHAT) to mothers with infants to prevent childhood obesity. BMC Public Health 17, 79. 28088203 10.1186/s12889-016-4005-x PMC5237545

[17] Ekambareshwar M , Mihrshahi S , Wen LM et al. (2018) Facilitators and challenges in recruiting pregnant women to an infant obesity prevention programme delivered via telephone calls or text messages. Trials 19, 494. 30219067 10.1186/s13063-018-2871-5 PMC6139132

[18] NSW Health Government (2019) Blue Book. https://www.health.nsw.gov.au/kidsfamilies/MCFhealth/Pages/child-blue-book.aspx#:∼:text=Summary,copy%20of%20the%20Blue%20Book (accessed August 2022).

[19] Dennedy MC & Dunne F (2013) Macrosomia: defining the problem worldwide. Lancet 381, 435–436. 23290492 10.1016/S0140-6736(12)62090-X

[20] Centre for Epidemiology and Research (2007) 2006 Report on Adult Health from New South Wales Population Health Survey. https://www.health.nsw.gov.au/surveys/adult/Publications/adults-06.pdf (accessed September 2022).

[21] NSW Government (2020) New South Wales Population Health Surveys. https://www.health.nsw.gov.au/surveys/Pages/default.aspx#:∼:text=Adult%20Population%20Health%20Survey&text=The%20NSW%20Adult%20Health%20Survey,February%20and%20December%20each%20year (accessed September 2022).

[22] Riley M , Rutishauser I & Webb K (2001) Comparison of Short Question with Weighted Dietary Records. Canberra: Australia Food and Nutrition Monitoring Unit, Commonwealth Department of Health and Aged Care.

[23] Wen LM , Simpson JM , Rissel C et al. (2013) Maternal “junk food” diet during pregnancy as a predictor of high birthweight: findings from the healthy beginnings trial. Birth 40, 46–51. 24635424 10.1111/birt.12028

[24] Department of Health and Aged Care (2021) Nutrition Advice during Pregnancy. https://www.health.gov.au/sites/default/files/documents/2021/06/nutrition-advice-during-pregnancy.pdf (accessed September 2022).

[25] NSW Government (2015) NSW Population Health Survey 2015 Questionnaire. https://www.health.nsw.gov.au/surveys/adult/Documents/questionnaire-2015.pdf (accessed September 2022).

[26] Li C , Zeng L , Wang D et al. (2019) Effect of maternal pre-pregnancy BMI and weekly gestational weight gain on the development of infants. Nutr J 18, 6. 30674315 10.1186/s12937-019-0432-8 PMC6345052

[27] Coelho NLP , Cunha DB , Esteves APP et al. (2015) Dietary patterns in pregnancy and birth weight. Rev Saude Publica 49, 62. 26398873 10.1590/S0034-8910.2015049005403 PMC4617437

[28] Englund-Ögge L , Brantsæter AL , Haugen M et al. (2012) Association between intake of artificially sweetened and sugar-sweetened beverages and preterm delivery: a large prospective cohort study. Am J Clin Nutr 96, 552–559. 22854404 10.3945/ajcn.111.031567 PMC3417215

[29] Mikkelsen TB , Orozova-Bekkevold I , Knudsen VK et al. (2006) Association between fruit and vegetable consumption and birth weight: a prospective study among 43,585 Danish women. Scand J Public Health 34, 616–622. 17132595 10.1080/14034940600717688

[30] Malek L , Umberger W , Makrides M et al. (2016) Adherence to the Australian dietary guidelines during pregnancy: evidence from a national study. Public Health Nutr 19, 1155–1163. 26228526 10.1017/S1368980015002232 PMC10271122

[31] Australian Institute of Health and Welfare (2020) Gestational Diabetes. https://www.aihw.gov.au/reports/diabetes/diabetes/contents/how-many-australians-have-diabetes/gestational-diabetes (accessed January 2023).

[32] Menard V , Sotunde OF & Weiler HA (2020) Ethnicity and immigration status as risk factors for gestational diabetes mellitus, anemia and pregnancy outcomes among food insecure women attending the Montreal diet dispensary program. Can J Diabetes 44, 139.e1–145.e1. 31427254 10.1016/j.jcjd.2019.05.004

[33] NSW Ministry of Health (2018) NSW Mothers and Babies Report 2017. https://www.health.nsw.gov.au/hsnsw/Publications/mothers-and-babies-2017.pdf (accessed September 2022).

[34] Fan C , Huang T , Cui F et al. (2015) Paternal factors to the offspring birth weight: the 829 birth cohort study. Int J Clin Exp Med 8, 11370–11378. 26379952 PMC4565335

[35] Hegaard HK , Petersson K , Hedegaard M et al. (2010) Sports and leisure-time physical activity in pregnancy and birth weight: a population-based study. Scand J Med Sci Sports 20, e96–e102. 19422639 10.1111/j.1600-0838.2009.00918.x

[36] Ruifrok AE , Althuizen E , Oostdam N et al. (2014) The relationship of objectively measured physical activity and sedentary behaviour with gestational weight gain and birth weight. J Pregnancy 2014, 567379. 25309754 10.1155/2014/567379 PMC4189770

[37] Both MI , Overvest MA , Wildhagen MF et al. (2010) The association of daily physical activity and birth outcome: a population-based cohort study. Eur J Epidemiol 25, 421–429. 20437195 10.1007/s10654-010-9458-0 PMC2896625

[38] Reid EW , McNeill JA , Alderdice FA et al. (2014) Physical activity, sedentary behaviour and fetal macrosomia in uncomplicated pregnancies: a prospective cohort study. Midwifery 30, 1202–1209. 24861673 10.1016/j.midw.2014.04.010

[39] Department of Health and Aged Care (2021) Physical Activity and Exercise for Pregnancy. https://www.health.gov.au/topics/physical-activity-and-exercise/pregnancy#limiting-time-sitting-and-lying-down (accessed January 2023).

[40] Klebanoff MA & Yip R (1987) Influence of maternal birth weight on rate of fetal growth and duration of gestation. J Pediatr 111, 287–292. 3612405 10.1016/s0022-3476(87)80089-6

[41] Goldstein RF , Abell SK , Ranasinha S et al. (2017) Association of gestational weight gain with maternal and infant outcomes a systematic review and meta-analysis. JAMA 317, 2207–2225. 28586887 10.1001/jama.2017.3635 PMC5815056

[42] Macdiarmid J & Blundell J (1998) Assessing dietary intake: who, what and why of under-reporting. Nutr Res Rev 11, 231–253. 19094249 10.1079/NRR19980017

